# Executive Functions of Divers Are Selectively Impaired at 20-Meter Water Depth

**DOI:** 10.3389/fpsyg.2017.01000

**Published:** 2017-06-20

**Authors:** Fabian Steinberg, Michael Doppelmayr

**Affiliations:** Department of Sport Psychology, Institute of Sport Science, Johannes Gutenberg University MainzMainz, Germany

**Keywords:** nitrogen narcosis, inert gas narcosis, water immersion, hyperbaric environment, SCUBA, cognition, human performance

## Abstract

Moving and acting underwater within recreational or occupational activities require intact executive functions, since they subserve higher cognitive functions such as successful self-regulation, coping with novel situations, and decision making; all of which could be influenced by nitrogen narcosis due to elevated partial pressure under water. However, specific executive functions that could provide a differentiated view on humans’ cognitive performance ability have not yet been systematically analyzed in full-water immersion, which is a research gap addressed within this approach to contribute to a better understanding of nitrogen narcosis. In this study, 20 young, healthy, and certified recreational divers participated and performed three different executive-function tests: the Stroop test (Inhibition), the Number/Letter test (Task switching), the 2-back test (Updating/Working memory), and a simple reaction time test (Psychomotor performance). These tests were performed once on land, at 5-meter (m) water depth, and at 20-meter (m) water depth of an indoor diving facility in standardized test conditions (26°C in all water depths). A water-proofed and fully operational tablet computer was used to present visual stimuli and to register reaction times. Performance of the simple reaction time test was not different between underwater and land testing, suggesting that reaction times were not biased by the utilization of the tablet in water immersion. Executive functions were not affected by the shallow water immersion of 5-m water depth. However, performance scores in 20-m water depth revealed a decreased performance in the incongruent test condition (i.e., an index of inhibitory control ability) of the Stroop test, while all other tests were unaffected. Even though only one out of the three tested cognitive domains was affected, the impairment of inhibitory control ability even in relatively shallow water of 20-m is a critical component that should be considered for diver’s safety, since inhibition is required in self-control requiring situations where impulsive and automatic behavior must be inhibited. Our interpretation of these selective impairments is based on a discussion suggesting that different neural networks within the central nervous system, which process specific executive functions, are affected differently by nitrogen narcosis.

## Introduction

Breathing air at increased ambient pressure can provoke inert gas narcosis (IGN) that affects the human nervous system, including alterations of cognitive functions, motor control, and mood states (Bennett and Rostain, 2003; Clark, 2015). Nitrogen narcosis is the most prominent form of IGN in recreational divers and it is thought that associated cognitive and motor impairments can increase the risk for incidents and reduce working performance (Kneller et al., 2012). In unexpected and dangerous situations, being it in underwater or other exceptional environments, an intact executive control system is necessary to guarantee not only a fast but also a correct decision. Executive abilities allow quickly adaptation to new requirements by shifting the mind set while simultaneously inhibiting inappropriate behaviors (Jurado and Rosselli, 2007). Such abilities might be necessary, e.g., in out-of-air situations in which it is required to quickly adapt to the unexpected situation by shifting attention to the new situation and the urge to ascent uncontrolled to the surface must be inhibited while evaluating the action possibilities. Moreover, information from internal sensory systems (e.g., urge to breath) along changing information from the environment (e.g., current depth, buoyancy status, position of the buddy) must be held in working memory, while constantly updating these information due to permanently changing states. Although several executive functions have been reported and different classifications and concepts have been postulated (Banich et al., 2000; Jurado and Rosselli, 2007; Banich, 2009; Diamond, 2013), it is thought that executive functions serve as the basis for higher cognitive control processes such as decision making and self-regulation and are thus extremely important for human performance and safety in extreme environments. However, specific executive functions (further definition on what is meant by specific is provided below) have not been investigated so far at water depths that are relevant for recreational divers, even though it would be of particular interest for diving safety and for further understanding of narcosis to reveal whether and to which degree specific executive control processes are impaired due to nitrogen narcosis.

Besides the problem that the existing knowledge regarding human performance and cognitive impairments under water is controversial and performance outcomes possibly biased by emotional, situational and other factors (c.f. Baddeley, 2000; Freiberger et al., 2016; Lafere et al., 2016), little attention has been paid to shallower water depths (Petri, 2003), i.e., pressure less or equal than 4–5 absolute atmosphere pressure (ATA; 1 ATA corresponds to 760 mmHg or to 1.01325 bar), which is commonly thought as the critical threshold to clearly identify nitrogen narcosis (Braubakk and Neuman, 2003; Clark, 2015). More specifically, detrimental effects on cognition and psychomotor performance, measured by a computer, as well as by means of objective measures of brain cortical arousal, have been detected already at 1.5–5 ATA in the hyperbaric chamber and in real-water immersion (Poulton et al., 1964; Petri, 2003; Balestra et al., 2012; Dalecki et al., 2012, 2013; Freiberger et al., 2016; Lafere et al., 2016; Germonpré et al., 2017), and changes in brain cortical arousal were even reported to be present 30-min after surfacing (Balestra et al., 2012; Lafere et al., 2016; Germonpré et al., 2017) Other reports suggest that a considerable amount of human performance decrements during diving might be attributed to open-water situations (Nevo and Breitstein, 1999; Baddeley, 2000). However, shallow-water immersion in controlled test conditions (i.e., not in open water) affects human cognitive processing (e.g., psychomotor speed and mental rotation ability) even when psychological factors (e.g., anxiety and mood) are eliminated as possible biasing factors (Dalecki et al., 2012, 2013). However, whether specific executive functions are impaired shallower than the critical threshold has not yet been studied.

Another important methodological issue is the kind of performance testing since cognitive performance has been tested across different studies with various test methods, definitions, task demands, and task complexities in different depths and test conditions, which often lead to ambiguity of the underlying cognitive concept under investigation. More specifically, most tests deployed in the past, e.g., classical neuropsychological tests such as card sorting, visual search, trail making tests, and multitasking and mathematical tests, tap into multiple-cognitive resources and involve non-executive processes (Miyake et al., 2000; Friedman et al., 2008; Miyake and Friedman, 2012). Recent advancements in cognitive psychology and neuroscience, however, suggest that executive processes, which underlie higher cognition, could be measured relatively isolated with more specificity by distinguishable executive functions (EF) tests. Those are thought of being more sensitive to alterations in brain functions and psycho-pathological states compared to classical neuropsychological tests (Miyake et al., 2000; Friedman et al., 2008; Miyake and Friedman, 2012). It is assumed that EFs comprise a set of lower-level cognitive processes necessary for successful self-regulation, coping with novel situations, complex planning, and decision making that are primarily localized but not exclusively in the prefrontal cortex of the brain (Banich, 2009; Miyake and Friedman, 2012; Snyder et al., 2015). Three executive functions measured by specific tests that were frequently used in the past served as the basis for an influential and relatively new cognitive model, the unity-diversity model, which assumes that executive functions share some commonalities but are nevertheless separable and represent their unique functions (Miyake et al., 2000). This idea is supported by neurophysiological evidence that defines the existence of a superior cognitive control network, which is reflected by common neural activation patterns and by domain-specific activation patterns (Niendam et al., 2012). Based on this model (Miyake et al., 2000; Friedman et al., 2008; Miyake and Friedman, 2012), the commonalities and behavioral differences are characterized by three core aspects of cognitive control: the ability to update relevant information in the working memory, to switch between different tasks and rule sets, and to inhibit responses to dominant, prepotent stimuli; all of which can be measured relatively isolated by specific test procedures (Snyder et al., 2015) and which are thought to form the basis for higher functions such as problem solving, reasoning, and planning (Diamond, 2013). There are different tests available to capture those specific executive functions by computer-based procedures including reaction time and error scores measures. Three of the most frequently used tests, which were used within the present approach, are the Stroop test as an index of the inhibitory control ability, the n-back test as an index of working memory capacity and the Number/Letter test as an index of the so called set-shifting ability (e.g., Miyake et al., 2000; Diamond, 2013). However, for all the tests, numerous variants for different research or clinical demands exist.

As outlined above, specific EFs have not been investigated in water immersion at depths that are relevant for recreational divers (i.e., <5 ATA), which is a research gap that is addressed with the current experiment. We argue that measuring EFs provide advantages in measuring cognitive performance in real underwater conditions compared to classical neuropsychological tests or other tasks that tap into multiple and non-cognitive processes. They are well described in terms of their neural substrate; they are frequently used, clearly defined, provide good sensitivity, and specificity, use scant testing time, and provide objective measurers such as reaction time and error rates (if measured by a computer); which are all aspects that make the tests ideally suited for investigating cognition in environmental circumstances with restricted test possibilities such as underwater. The first aim of the present approach was, therefore, to investigate the three core EFs in real water immersed conditions from the unity-diversity perspective with the purpose of providing a differentiated view of underwater cognitive performance. The second and complementary aim was to increase the ecological validity (compared to hyperbaric chamber testing) by analyzing cognitive performance computer based and in real-water immersion while holding test conditions standardized and controlled.

Therefore, we questioned (i) whether we could identify performance decrements of executive functions elicited by relatively shallow water immersion of 5- and 20-meter (m) fresh water depth and (ii) whether a detailed and separated analysis of specific executive function reveals cognitive deteriorations more differentiated than other tasks that activate multiple executive control processes.

## Materials and Methods

### Participants

The requirement for participating in this study was a valid SCUBA (self-contained underwater breathing apparatus) diving license per European norm EN 14153-2, a valid medical fitness certificate, and a minimum of 10 dives, which at least two dives had to be deeper than 20-m water depth. Twenty qualified divers (4 females) aged 30 ± 8.7 years having a mean self-stated diving experience of 349 ± 516 (range was between 10 and 2000; and the median was 61) logged dives participated in the present experiment. Prior to the experiment, all participants were fully informed of the purpose of the study and filled an informed consent form. The test protocol followed the rules of the Helsinki declaration and was pre-approved by the ethics committee of the Deutsche Gesellschaft für Psychologie.

### Measurements of Cognitive Functions, Psychomotor Speed, Heart Rate, and Ventilation

**FIGURE 1:**
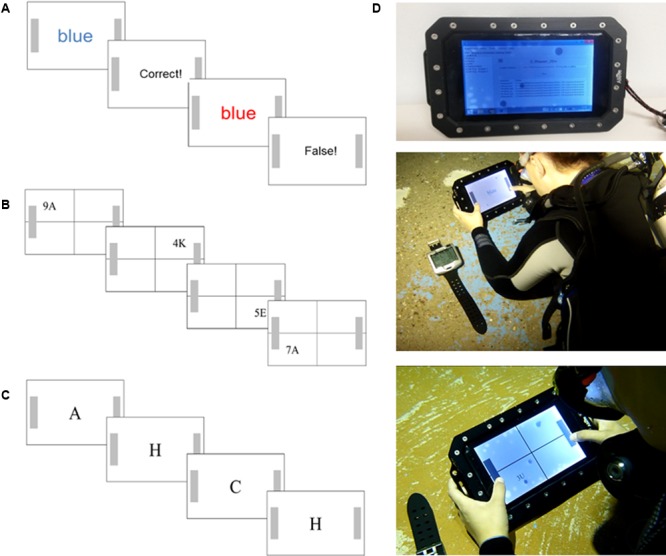
Test sequence and test setup: Example stimuli sequence of **(A)** the Stroop test, **(B)** the Number/Letter Test, and **(C)** the 2-back test. **(D)** Photography of the underwater tablet computer, the diving computer, and a diver performing the tests underwater. More details are presented within the text.

For all tests, we used a waterproof Windows based tablet computer (Alleco^®^, Finland) with a touchscreen being seven inches and framed in an aluminum case. Through a special liquid within a leaf that is put over the screen, the touch screen was fully operational underwater. Thus, this tablet computer served as the visual stimuli presentation within cognitive tests and as the stimulus response device registering finger presses at the touchscreen at specified regions (more details are provided below). The advantage of this tablet is that the fingers were placed on the screen, and finger presses were the same in water immersion and on land, i.e., due to the incompressible liquid between the screen and the leaf, the forces required to press the button were the same in all conditions. Thus, water viscosity should not affect reaction-time measurements, which was confirmed with an additional reaction time test (see results, **Figure 1**). For stimuli delivery and response measures, we used the software Presentation (Neurobehavioral Systems^®^, United States). Every four seconds, heart rate, and tank air pressure were recorded and digitally stored with a commercially available diving computer (Galileo Sol; Uwatec^®^, Swiss). Heart rate was averaged only for the time frame in which the tests were performed underwater (about 10 min). We additionally estimated the pressure adjusted amount of air (i.e., corrected to normobaric condition) breathed within one minute (i.e., l/min) by using the gas-pressure change in the tank within a 1-min period. This ventilation was than averaged for the complete time frame of executing the cognitive tests. For the baseline measures, no heart rate and ventilation were monitored due to the limited usability of the diving computer to measure without any elevated atmospheric pressure.

Based on Miyake et al. (2000), three executive function tests that are thought to form the core basis of higher cognitive functions, were used to measure inhibitory control (Inhibition), task shifting (Shifting), and working memory (Updating). All tasks were modified in terms of their application on a tablet computer and restricted time for testing in 20-m water depth (i.e., to avoid a decompression dive and consider limited air supply).

#### Inhibition

This test is a modified and computerized short version of the classical Color-Word Stroop test (Stroop, 1935), which is thought to reflect inhibitory or interference control ability (e.g., MacLeod, 1991; Friedman and Miyake, 2004). As depicted in **Figure 1A**, the test consists of a congruent and an incongruent color-word condition. In the congruent condition, presented words [German: blau and rot (blue and red)] were printed in the same color as the semantic meaning of the word (i.e., the word blue was printed in blue color). In the incongruent condition, the presented word was not in the same color as the semantic meaning of the word (e.g., the word blue was printed in red colors). In this incongruent color-word condition, the prepotent response to react to the written word must be inhibited since it is required to react to the color of the ink. This results in an interference effect reflected by increased reaction time scores (compared to the congruent condition) and it is thought to index the ability to inhibit a dominant response (e.g., Aron, 2007). The test consisted of a presentation of 64 words, 32 were congruent, and the other 32 were incongruent, which were presented randomly. Each word was presented for a maximum of 2500 ms (if not responded) and followed by feedback (correct or false response) and the next target word was presented with an inter stimuli interval (ISI) between 800 and 1600 ms. Participants were instructed to press the right button when the word meaning corresponded to the color red and the left button if not corresponding and vice versa for the blue color. Reaction times were computed by calculating the mean value and excluding reaction times lower than 100 and higher than 2000 ms. Additionally, reaction times were separately calculated for the congruent and incongruent trials, and the “Stroop effect” was calculated by subtracting the mean reaction time of the congruent trials from the incongruent trials. Wrong responses (errors) for both stimuli categories (congruent and incongruent) were also counted.

#### Shifting

To measure the executive function shifting ability, a modified and shorter version of the Number/Letter task (Rogers and Monsell, 1995) was used, which consisted of presenting Number/Letter pairs in one of four quadrants at the tablet’s screen in a clockwise direction beginning at the upper-left quadrant (see **Figure 1B**). When the Number/Letter pair was presented in the upper two quadrants, participants had to decide whether the number was uneven or even and had to press either the left (even) or right (uneven) button, respectively. When the Number/Letter pair was presented in the bottom quadrants, they had to decide whether the letter was a consonant or a vowel and had to press the left button for the consonant or the right bottom for the vowel. Thus, the tests contained two trials (i.e., Number/Letter pairs) in which the task rule was constant (i.e., upper quadrants) and two trials in which the task rule switched (i.e., from the upper right to the bottom right quadrant and from the bottom left to the upper-left quadrant). It is thought that the switch between the task rules provokes increased reaction times due to the mental shift from one rule to the other, i.e., the shifting ability (Rogers and Monsell, 1995). In total, 64 pairs were presented, and each pair was presented for a maximum for 3000 ms, the ISI was 300–500 ms, and the letter-number pairs were randomly presented. Reaction times were computed by calculating the mean value and excluding reaction times lower than 100 and higher than 2000 ms. Additionally, reaction times were calculated separately for the conditions having no switch in the task rule (No-Switch) and for the condition with a switch in the task rule (Switch). The “Switch-Cost” was computed by subtracting the mean reaction times of the No-Switch trials from the Switch trials. Wrong responses (errors) for both stimuli categories (Switch and No-Switch) were also counted.

#### Updating

A modified and short version of the 2-back task was used to measure the executive function of memory updating (Kirchner, 1958). As depicted in **Figure 1C**, at the screen, a row of letters was presented consecutively whereas each letter was presented solely for 500 ms and replaced by another letter with an ISI of 1000 ms. Participants were required to indicate by button press (right press for right handers and left press for left handers) when a letter was presented that has already been presented two letters previously. If the displayed letter was not presented two letters previously, no reaction was required. Thus, this test requires to actively maintain two letters in the working memory while continuously updating the working memory with a new letter, i.e., the first letter in the sequence has to be removed from the memory and must be replaced by a new letter (e.g., Kane et al., 2007). There were 60 letters presented in the test, which 20 letters were target letters and letter presentation sequences were different in each condition. The 2-back task mean reaction time was computed for all successfully identified letters, while the amount of false alarms (i.e., button press although no target letter) and the missed targets (i.e., no bottom press although a target letter) were counted.

#### Psychomotor Function

A simple one-choice reaction time task was used to measure psychomotor function. A red-small square was presented in the middle of the screen for a maximum of 500 ms. After the button response was performed as fast as possible, the next square was randomly presented with an ISI between 800 and 1600 ms. There were 32 squares presented, and participants were required to press the target button at the screen with the right thumb (left thumb for left handers). Reaction times were computed by calculating the mean value and excluding reaction times lower than 100 and higher than 1000 ms.

### Experimental Procedure

The experiment occurred in an indoor-diving facility. The water temperature at any depth was 26°C, and the maximum water depth was 20 m with excellent visibility. Participants, after arriving at the test location, were asked to fill in questionnaires regarding their diving experience and other anthropometric data. Then, the cognitive tests were explained by the investigator and a test trial including all tests, and the full amount of test stimuli was performed by each participant to minimize learning effects from baseline testing to the first underwater test condition.

The subsequent experimental sequence was as follows: Before the baseline land measures were performed, the diving equipment consisting of standard SCUBA diving equipment (buoyancy control device, breathing regulator, 10-liter tank, and 3-mm neoprene suit, mask, fins, and diving computer) were mounted. Baseline measures were performed in a seated and comfortable position and participants wore the diving mask, breathed through the regulator, and wore earmuffs. All cognitive tests were performed with the tablet computer and in the same sequence: The order was the reaction time test (RT-Test) followed by the Stroop test, the Number/Letter test, and the 2-back test. Before each test, a short familiarization trial (8 stimuli) was provided to be certain of task understanding and 30 s breaks were included between each test. After baseline measurements, participants were equipped with the diving gear and the belt for heart-rate measurements. All 20 participants performed the tests in the same sequence and the same rest breaks once on a platform at 5-m water depth and once at 20-m depth in an almost lying position (**Figure 1D**). There were 11 participants pseudo-randomly assigned to start at 5 m and 9 others started at 20 m. This randomization was chosen to average out possible learning effects, that could occur between the baseline measure and the first test either at 5-m or at 20-m water depth. After the descent either to 5 or to 20 m and before task execution, participants could freely swim in a slow pace underwater for 5 min to ensure familiarization with the environment. This procedure resulted in a dive timeline for the group that started at 5 m (referred to as 5–20 m group hereafter) in starting the first test about seven min. (1 min. descent, 5 min. fin-swimming, one min. task preparation) after leaving the surface, 10 min of testing at 5 m, about two min. descent to the 20 m depth, 5 min. of fin-swimming, 1 min. task preparation, 10 min. of testing, and 2 min. of ascent and 3 min. safety stop at 3 m (i.e., the full dive lasted about 40 min. for each participant). The same procedure was performed by the group that started at 20 m (referred to as 20–5 m group hereafter) with the difference that the descent to 20 m took about 2 min. more, and the safety stop was included in the 5 min. fin-swimming at 5 m.

### Statistical Analysis

All variables were checked for a violation of normal distribution using the Shapiro–Wilks test. In the case of normally distributed variables, mixed ANOVAs with a between factor group (5–20 m group/20–5 m group) and with repeated measures on the factor condition (Baseline/5 m/20 m) were performed to reveal any influence of water immersion, increased pressure in 20-m depth and to observe whether the time of exposure (i.e., different effects between the group that performed the tasks beginning at 5 m and the group beginning at 20 m) had any effect on task performance. Significant results were further explored by Bonferroni-corrected pairwise comparisons (*t*-tests). Effect sizes were estimated according to Cohen (1988) by partial eta-squares ($\eta_{p}^{2}$), where $\eta_{p}^{2}$ > 0.01 indicates a small effect, $\eta_{p}^{2}$ > 0.06 indicates a medium effect and $\eta_{p}^{2}$ > 0.14 indicates a large effect. In the case of non-normally distributed variables, Friedman tests with the same factor for the condition effects were performed, and Mann–Whitney-*U* tests for all variables were performed between groups. Since heart rate and ventilation were only recorded in water immersion and data were normally distributed, dependent *t*-tests between 5- and 20-m conditions for both variables were used.

## Results

The *t*-tests for heart rate at 5 m (83.29 ± 16.82 bpm) and ventilation (10.51 ± 3.12 l/min) revealed that they were not significantly (both *p* > 0.05) different to the same measurements at 20 m (79.02 ± 12.49 bpm; 10.18 ± 2.60 l/min), which indicated that participants were not differently physically active in both test conditions. Psychomotor speed, as tested by the RT-test at the beginning of the experiment was not significantly different between test conditions [*F*(2,36) = 2.18; *p* = 0.12], although reaction times slowed underwater by about 10 ms at 5 m and 12 ms at 20 m. There were no group [*F*(1,18) = 0.41; *p* = 0.52] and no condition^∗^group interaction effects [*F*(2,36) = 0.059; *p* = 0.94].

**FIGURE 2:**
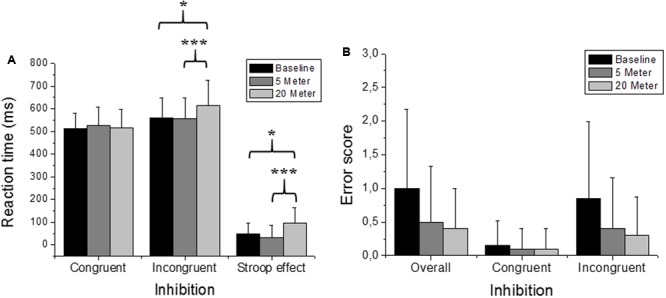
Stroop task performance (i.e., inhibition). Depicted are mean reaction times and the mean errors. **(A)** Stroop task performance of the congruent and the incongruent stimuli and the resulting Stroop effect. **(B)** “Overall” represents all errors independent of the stimuli, while the separated errors for the congruent and incongruent stimuli are depicted as well. Values representing the arithmetic mean and error bars indicate the corresponding standard deviations. ^∗^Denotes *p* < 0.05 and ^∗∗∗^*p* < 0.001.

**FIGURE 3:**
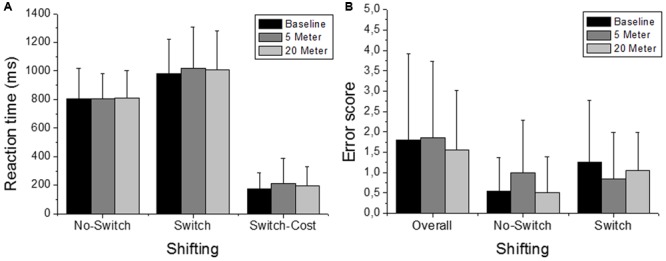
Number/Letter task performance (i.e., shifting). Depicted are reaction times and the error scores. **(A)** Reaction times of the No-Switch trials and the Switch trials and the corresponding Switch-Costs. **(B)** “Overall” includes the errors that occurred in the complete test and separated errors for the different trials (No-Switch and Switch) are depicted separately as well. Values representing the arithmetic mean and error bars indicate the corresponding standard deviations.

**FIGURE 4:**
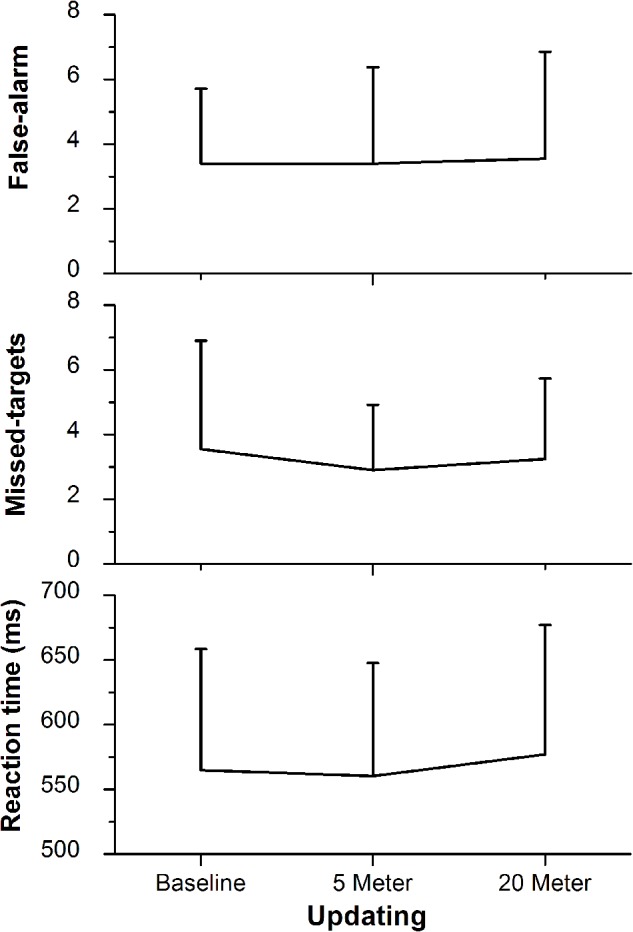
2-back task performance (i.e., updating) with the lower panel representing the reaction time, the middle panel representing the amount of missed target letters, and the upper panel representing the false-alarm rate. Values representing the arithmetic mean and error bars indicate the corresponding standard deviations.

**Figures 2, 3, 4** illustrate the separated analysis of the three executive function tests with **Figures 2A,B**, depicting the Stroop test performance (i.e., inhibitory-control ability). In addition, **Table 1** depicts all variables of the Stroop task performance separated for each group. Reaction times of the simple condition (i.e., congruent stimuli) were not affected by water immersion or by increased water depth (ANOVA condition effect was *F*(2,36) = 0.46; *p* = 0.63). There was also no group [*F*(1,18) = 0.20; *p* = 0.89] and no condition^∗^group interaction effect [*F*(2,36) = 0.49; *p* = 0.61]. However, ANOVA for the reaction times of the incongruent trials yielded a highly significant main effect with high effects sizes [*F*(2,36) = 7.92; *p* = 0.001; $\eta_{p}^{2}$ = 0.30]. Moreover, groups did not differ in task performance [*F*(1,18) = 0.003; *p* = 0.95] and there was no interaction effect between condition and group [*F*(2,36) = 0.45; *p* = 0.64] *Post hoc* comparisons revealed that reaction times were higher (i.e., slower response) in 20-m water depth compared to the dry baseline test (*p* = 0.037; +51 ms, and 9.2% slower) and to the 5-m water immersion condition (*p* = 0.001; +55 ms, and 9.1% slower). No differences were detected between the 5-m condition and baseline condition (*p* > 0.05). The pure measure of inhibitory control (i.e., the Stroop effect) confirmed these findings, since the ANOVA main effect [*F*(2,38) = 9.74; *p* < 0.001; $\eta_{p}^{2}$ = 0.39] was highly significant. Again, no group effect [*F*(1,18) = 0.12; *p* = 0.72] and no condition^∗^group interaction [*F*(2,36) = 1.90; *p* = 0.16] emerged. Accordingly, *post hoc* measures revealed that inhibition at 20 m was significantly inferior to the baseline measure (*p* = 0.014) and to the 5-m depth (*p* < 0.001), while the measurements at 5 m compared to baseline were not different (*p* > 0.05). Friedman’s ANOVA for the error scores of the congruent trials (χ^2^ = 0.40; *p* = 0.81), the incongruent trials (χ^2^ = 2.91; *p* = 0.23) and for the overall error (χ^2^ = 4.49; *p* = 0.10) detected no statistical differences in the amount of error between test conditions. Moreover, we found no differences between the groups in all Stroop task related error scores (all *p* > 0.05).

**Table 1:** Reaction times and error scores of the Stroop task separated by condition and experimental group.

Reaction times of the Stroop test (Inhibition)									
	**Congruent**	**Congruent**	**Congruent**	**Incongruent**	**Incongruent**	**Incongruent**	**Stroop effect**	**Stroop**	**Stroop**
**Group**	**Baseline**	**5-m**	**20-m**	**Baseline**	**5-m**	**20-m**	**Baseline**	**effect 5-m**	**effect 20-m**
5–20 m	513.7 ± 82.6	519.8 ± 85.3	524.1 ± 94.7	564.1 ± 109.8	559.9 ± 111.1	603.6 ± 138.2	50.3 ± 59.1	40.4 ± 41.8	79.6 ± 72.6
20–5 m	509.3 ± 46.6	530.5 ± 81.2	504.6 ± 68.6	558.1 ± 46.0	553.2 ± 69.6	623.4 ± 81.4	48.7 ± 29.8	22.6 ± 67.9	118.7 ± 54.6
**Error scores of the Stroop test (Inhibition)**									
	**Congruent**	**Congruent**	**Congruent**	**Incongruent**	**Incongruent**	**Incongruent**	**Overall**	**Overall**	**Overall**
**Group**	**Baseline**	**5-m**	**20-m**	**Baseline**	**5-m**	**20-m**	**Baseline**	**5-m**	**20-m**

5–20 m	0.18 ± 0.40	0.09 ± 0.30	0.18 ± 0.40	0.36 ± 0.67	0.18 ± 0.40	0.27 ± 0.46	0.54 ± 0.68	0.27 ± 0.46	0.45 ± 0.52
20–5 m	0.11 ± 0.33	0.11 ± 0.33	0.00 ± 0.00	1.44 ± 1.33	0.66 ± 1.00	0.33 ± 0.70	1.55 ± 1.42	0.77 ± 1.09	0.33 ± 0.70

*Depicted are means and standard deviations.*

The ability to switch between task rules (i.e., shifting ability) was not influenced by shallow-water immersion (5 m) or by deeper-water immersion (20 m), since in all three, ANOVAs condition effects on the reaction time of the variables Switch, No-Switch, and Switch-Cost were not significant (all three had *p* > 0.05; **Figure 3A**). There were also no group or condition^∗^group interaction effects for all variables (all *p* > 0.05; **Table 2**). Additionally, Friedman’s ANOVA could not detect significant differences in the amount of error between test conditions (all three had *p* > 0.05; **Figure 3B**) and *U* tests detected no significant differences between groups (*p* > 0.05). The same accounts for the ability to update information in working memory as measured by the 2-back test (Updating ability). Neither the condition effect of the ANOVA for the 2-back’s reaction time was significant nor were the group or condition^∗^group interaction significant (*p* > 0.05; **Figure 4** and **Table 3**). Moreover, Friedman tests for the false-alarm and the missed-targets parameters revealed no significant influences of water immersion to 5 or 20 m water depth compared to land (both *p* > 0.05, **Figure 4**) and there were no differences between groups (all *p* > 0.05).

**Table 2:** Reaction times and error scores of the Number/Letter task separated by test condition and experimental group.

Reaction times of the Number/Letter task (Shifting)									
	**No-Switch**	**No-Switch**	**No-Switch**	**Switch**	**Switch**	**Switch**	**Switch-Cost**	**Switch-Cost**	**Switch-Cost**
**Group**	**Baseline**	**5-m**	**20-m**	**Baseline**	**5-m**	**20-m**	**Baseline**	**5-m**	**20-m**
5–20 m	822.6 ± 259.3	837.8 ± 203.3	804.3 ± 230.2	992.4 ± 275.3	1048.6 ± 308.1	986.9 ± 281.7	169.7 ± 129.2	210.7 ± 149.4	182.5 ± 119.8
20–5 m	782.1 ± 157.0	766.5 ± 140.5	817.2 ± 143.9	967.5 ± 205.6	983.0 ± 271.5	1032.1 ± 276.2	185.4 ± 88.8	216.5 ± 208.2	214.8 ± 151.4
**Error scores of the Number/Letter task (Shifting)**									
	**No-Switch**	**No-Switch**	**No-Switch**	**Switch**	**Switch**	**Switch**	**Overall**	**Overall**	**Overall**
**Group**	**Baseline**	**5-m**	**20-m**	**Baseline**	**5-m**	**20-m**	**Baseline**	**5-m**	**20-m**

5–20 m	0.45 ± 0.93	1.09 ± 1.37	0.36 ± 0.92	1.36 ± 1.74	0.36 ± 0.67	0.90 ± 0.83	1.81 ± 2.52	1.45 ± 1.69	1.27 ± 1.42
20–5 m	0.66 ± 0.70	0.88 ± 1.26	0.66 ± 0.86	1.11 ± 1.26	1.44 ± 1.33	1.22 ± 1.09	1.77 ± 1.64	2.33 ± 2.06	1.88 ± 1.55

*Depicted are means and standard deviations.*

**Table 3:** Reaction times (RT) and error scores of the 2-back task separated by test condition and experimental group.

Reaction times and error scores of the 2-back task (Updating)									
	**RT**	**RT**	**RT**	**Missed-targets**	**Missed-targets**	**Missed-targets**	**False-Alarm**	**False-Alarm**	**False-Alarm**
**Group**	**Baseline**	**5-m**	**20-m**	**Baseline**	**5-m**	**20-m**	**Baseline**	**5-m**	**20-m**
5–20 m	567.5 ± 112.9	559.7 ± 99.9	578.9 ± 121.5	4.45 ± 4.18	3.81 ± 2.18	3.72 ± 2.57	3.09 ± 2.21	3.90 ± 3.04	3.72 ± 3.49
20–5 m	561.6 ± 69.1	561.2 ± 74.7	575.0 ± 72.7	2.44 ± 1.50	1.77 ± 1.09	2.66 ± 2.39	3.77 ± 2.53	2.77 ± 2.94	3.33 ± 3.24

*Depicted are means and standard deviations.*

## Discussion

The central purpose of the present approach was to investigate cognitive performance in shallow water immersion by using executive-function tests. To the best of our knowledge, this study is the first that measured specific executive functions at 20-m water depth. Separated analysis of the three specific executive functions revealed that only the inhibitory control ability, measured by the Stroop test, was influenced by the 20-m water depth, while the switching and updating abilities were not affected. More specifically, incongruent reaction times were increased without statistically significant changes in error-rates, which suggests that the participant’s cognitive system slowed and held accuracy constant, i.e., there is no substantial evidence for a change in strategic behavior in the speed-accuracy setting as has been observed in other studies (Fowler et al., 1985; Sparrow et al., 2000). However, descriptively at 20-m water depth error rates in the Stroop test decreased compared to baseline conditions, which suggests a slight change in the speed-accuracy setting, although not significant (*p* = 0.10 for overall error of the Stroop test). As depicted in **Table 2**, this decrease was more pronounced for those participants that started at 20-m water depth and less for the group that started at 5-m water depth, which points toward slight learning effects rather than changes in strategic behavior. However, given our experimental design, small sample size, and the respective analysis, we cannot completely exclude that a change in strategic behavior might have occurred.

The reaction-time task revealed that reaction times that are normally not affected at these pressure levels are not different in water immersion and on land, thus providing evidence that the use of the touch screen of the tablet computer did not systematically bias reaction time registrations in full water immersion and different depths. The experiment was conducted in a relatively safe environment with constant temperature and visibility, and heart rate and ventilation were at low rates and not different between experimental conditions. Thus, it is unlikely that anxiety or physical activity might have influenced task performance. Consequently, the biasing factors of open water that have often been reported have not influenced our findings. Nevertheless, caution is still needed, since the cognitive component of anxiety has been neglected in this approach, and it is still unclear whether and which kind of anxiety affects all or only some executive processes (Eysenck et al., 2007; Derakshan and Eysenck, 2009), a factor that warrants further investigation.

Generally, our findings agree with observations that cognitive performance deteriorates in water depth shallower than the usually accepted nitrogen narcosis threshold of 4 ATA (Poulton et al., 1964; Petri, 2003; Dalecki et al., 2012, 2013). Because those observations with respect to our study were either detected with specific tests measured with a computer in the pressure chamber (Petri, 2003) or with a computer in shallow water immersion (i.e., 5 m) (Dalecki et al., 2012, 2013), the necessity for investigating cognitive functions in real water immersion in combination with sensitive and specific computer-based tests is confirmed. However, it should also be noted that only two out of the three tested cognitive domains were influenced by 20-m, which suggests that central parts of the cognitive control system retained their full functionality. The most interesting finding was that the inhibitory control ability was significantly impaired in 20-m water depths by about 9%. The inhibitory control ability is strongly involved in behavior, which is important to the safety and accident prevention in extreme environments. According to Diamond (2013), inhibition involves the ability to control behavior, thoughts, and emotions in situations where strong internal predispositions must be overrun. A lack of inhibitory control capacity would allow us to be driven by environmental stimuli and internal emotions that pull us in uncontrolled and possibly dangerous directions. Inhibitory control enables individuals to choose how to react and how to behave (Diamond, 2013); thus, it is necessary and active, e.g., in underwater out-of-air situations where the internal drive to ascent uncontrolled to the surface must be controlled (i.e., inhibited). Therefore, it is an important observation and of practical value for diving safety that the inhibitory control ability is already impaired at water depth of 20 m.

However, although the data analysis did not yield any group or condition^∗^group interaction effects, it should be considered that our experimental paradigm might include that the effects are partially blurred due to learning/practice effects and different time of exposure to nitrogen partial pressure. If learning effects have occurred from baseline to the first measure then the overall decrease in performance of the first underwater condition (i.e., either 5 or 20-m) might underestimate real effects. Moreover, one group started the cognitive tests at 20-m water depth, while the other group started at 5-m water depth, i.e., the 5–20 m group, had one more chance to improve task performance due to learning. Thus, the reported effects at 20-m water depth of the 20–5 m group might underestimate the real effects and overestimate the effects for the 5–20 m group. Indeed, reaction times and error rates of the incongruent part of the Stroop test and the Stroop effect (cf. **Table 1**) indicate that this was the case for the Stroop test, and a similar pattern occurred in the Number/Letter task (cf. **Table 2**), i.e., slower performance and more errors for the 20–5 m group in 20-m compared to the 5–20 m group. Therefore, this discussion accounts in the same way not only for the Stroop test but also principally for the 2-back and the Number/Letter test. Nevertheless, there were no significant group and no condition^∗^group effects, which indicates that if learning effects occurred they were not substantial and did not bias our central conclusion. In addition, it is well known that time of exposure, independently from environmental condition, at 33 m water depth influence the critical flicker fusion frequency (CFFF), an objective measure of nitrogen narcosis, cortical arousal, and performance, with changes observable even up to 30 min. post-dive (Balestra et al., 2012; Hemelryck et al., 2013; Lafere et al., 2016). From this perspective one might argue that task performance of the 20–5 m group should have been deteriorated in 5-m, which was statistically not the case. The reason for this could either be related again to practice effects or mean that the executive function tests used in this approach were not sensitive enough to detect such longer lasting changes as has been done with the CFFF paradigm on 33-m dives. Alternatively, the pressure induced effects of nitrogen in 20-m were not enough to provoke those changes. However, due to the relatively small sample size within each group, despite any significance, caution on these factors is still needed and warrants more research, e.g., by a higher sample size, elevated water depths, combined behavioral (executive function testing), and more objective measures (CFFF) along a careful consideration of the testing order and exposure time.

Due to the absence of cognitive declines at only a water depth of 5 m, nitrogen narcosis is most likely the factor that has caused performance decrements due to elevated nitrogen partial pressure at 20 m. Our finding of selective impairments of executive functions is difficult to reconcile with theories of nitrogen narcotic effects on humans information processing capacity such as the general cognitive slowing model (Fowler et al., 1985) (i.e., different arousal), the evolutionary hypothesis (Kiessling and Maag, 1962), or the multiple processing model that has been recently proposed (Dalecki et al., 2013). Our results contrast with the latter study. Dalecki et al. (2013) found that the simple (congruent stimuli), but not the complex (incongruent stimuli), Stroop-task performance slowed already at 5-m water immersion. In contrast, only the incongruent condition was affected at 20-m water depth in the present study. One possible explanation, however, is that the error rates of the simple-test conditions were significantly affected in the Dalecki et al. (2013) study, while we found no substantial error-rate changes. Thus, a change in the speed-accuracy setting could have emerged in the simple condition of their study, while it has probably not occurred in the present approach.

### A Hypothetic Explanation of Selective Executive Function Impairments

Due to a lack of directly comparable studies and the difficulty to integrate our results within other models, we propose an alternative explanation of our results and several other recent findings of IGN effects by considering current insights in the mechanisms of IGN, neuroimaging approaches, and cognitive models of the human executive control system. We suggest that elevated nitrogen partial pressure due to water immersion of 20 m might have selectively impaired neuronal networks that are differently engaged in executive processes (Niendam et al., 2012). As has been shortly addressed in the introduction, meta-analysis indicates that different executive functions, subserving higher cognitive functions, involve the activation of a common frontal-cingulate-parietal-subcortical neural network. For each executive function, a unique activation pattern can be identified in distinction to other executive functions (Niendam et al., 2012), a finding which is supported by behavioral reaction time analysis using confirmatory factor analysis (Miyake et al., 2000). Considering this unity-diversity idea, our results suggest that 20-m water immersion does not generally affect the neural network, subserving all three EFs (i.e., the common areas) and rather exhibits its influence only onto specific neural structures that are unique for the Stroop task performance. The anterior cingulate cortex (ACC) is one brain structure that is strongly involved in conflict monitoring, as it is required in the incongruent (and not in the congruent) condition of the Stroop test but not necessarily in the 2-back and task-switching tests (Botvinick et al., 2001; van Veen et al., 2001; Owen et al., 2005; Mansouri et al., 2009). More specifically, the ACC is involved in detecting and resolving competing and simultaneously active representations (e.g., competing color and word meaning processing) and signals the need for attentional control to the dorsolateral prefrontal cortex (Kühn et al., 2016). In contrast, the switching between two tasks (i.e., successively as in the task-switching task) or within working memory tasks (i.e., as in the 2-back task), the involvement of this ACC function is minimized and other brain structures are involved (Dreher and Grafman, 2003; Owen et al., 2005).

Such different involvement of specific brain areas, however, cannot explain why they should be differently affected by nitrogen narcosis without considering new research that combines functional neuroimaging and the molecular basis of neurotransmitter within specific brain areas and insights from IGN mechanisms: There is accumulating evidence that inert gases act competitively at the level of cellular proteins, supporting a protein-binding theory, and thus modulate the regulation of the nigro-striatal pathway, which is involved in cognitive processes (Franks and Lieb, 1984; Abraini et al., 1998; review in Rostain et al., 2011). This pathway is primarily regulated by excitatory glutamatergic neurotransmitters and gamma amino butyric acid (GABA) inhibitory neurotransmission and responsible for dopamine levels in the striatium (Rostain et al., 2011). Furthermore, it has been shown that increased nitrogen pressure can decrease glutamate and dopamine levels and increase serotonin in rats (Vallée et al., 2009; Vallee et al., 2009). Therefore, these and other results show that nitrogen narcosis can disturb the glutamatergic pathways by GABA neurotransmission induced reduction of glutamate (Rostain et al., 2011), which in turn affects the functions of cortical structures. The ACC has been identified to accumulate glutamate faster than other brain regions when stimulated, e.g., within pain perception or cognitive tasks involving conflict monitoring such as in the Stroop task (Hutchison et al., 1999; Mullins et al., 2005; Taylor et al., 2015). Considering that a recent study found that pain perception is reduced during a simulated 50-m dive (Kowalski et al., 2012) and that ACC is strongly involved in pain perception (Rainville et al., 1997; Hutchison et al., 1999; Büchel et al., 2002), the ACC may be one brain area that is highly sensitive to neurotransmitter alterations induced by elevated nitrogen pressure. Complementary to this discussion, the step-by-step mechanism of anesthetic action could be a potential explanation for the differential effects of nitrogen on different executive functions (Colloc’h et al., 2007). They revealed that the anesthetic agents, xenon and nitrous oxide first bind to brain intracellular proteins with large hydrophobic cavities and disrupt functions of the targeted proteins, which in turn is sufficient to provoke symptoms of the early stage of anesthesia. Only the following step(s) with higher gas concentration involves gas-binding of smaller cavities (such as the NMDA receptor) leading to surgical anesthesia. It is thought that such a step-by-step mechanisms also accounts for other inhaled anesthetics and other receptors such as the GABA_A_ receptor (Colloc’h et al., 2007). Since it is also well known that different brain regions are differently deactivated during anesthesia, possibly due to different kinds and density of target receptors (Kaisti et al., 2003; Franks, 2008), this might also be responsible for differential onset and symptoms related to nitrogen narcosis. Thus, cognitive functions and other functions (e.g., pain perception) associated with ACC activity or possibly other sensitive brain structures might be influenced by narcosis earlier than other functions that are controlled by other brain structures. This in turn might also explain the selective impairment of executive functions within this approach and other studies that found selective impairments within a specific domain at a given hyperbaric environment.

However, although this hypothesis might be an interesting avenue for further research by applying behavioral tests that are clearly identified in terms of their neural substrate, it requires further methodological developments by establishing neuroscientific techniques within hyperbaric settings and/or ideally in real water immersed conditions. Clear inferences from behavioral measures to its neural substrate without neurophysiological measures should still be handled cautiously. Nevertheless, it appears that new avenues for IGN research in the field from a neuroscientific perspective is necessary by combining behavioral measures, recent developments in wireless electroencephalography (EEG) devices, and further objectives measures such as the CFFF. This might provide new insights into this field of research when technical advancements allow water-proof measurements, which has already been demonstrated with EEG (Schneider et al., 2014).

### Limitations

Since our approach is the first that addressed executive processes under “real-life” conditions, it has also some limitations. First, as has been already discussed, our experimental method cannot completely exclude practice effects, which should be excluded in future studies, e.g., by extensive learning prior to varying the experimental condition (e.g., Germonpré et al., 2017) or by an additional control group performing the same tests in the same and time-matched sequence without water immersion. In line with this, time of exposure should be additionally emphasized, e.g., by exposing subjects to only one water depth or by the application of between-subject instead of within-subject experimental designs. Second, the cognitive tests developed for this purpose were shortened to a minimal level of visual stimuli within each test to be certain that three different executive functions can be tested in a short time frame. Consequently, this might have provoked increased random noise in the reaction time outcome. Complementary to this limitation, the unity-diversity idea proposed by Miyake et al. (2000) was developed, besides other reasons, because of the so-called task-impurity problem, which means that even well-defined executive function tasks and scores derived from those tests still include systematic variance and measurement errors (i.e., random noise in the data) from non-executive processes (Miyake and Friedman, 2012). However, to account for this problem within real-life and extreme environmental conditions would require a fundamental other-experimental approach that is out of the scope of this study. Another limitation is that our environment was relatively safe, which makes it still difficult to transfer conclusions to varying conditions of open-water situations. Our experimental design was established to explore cognitive performance in different water depths compared to land by a computer, but it would be desirable for future studies to observe possible longer-lasting effects as has been shown by the use of the CFFF paradigm (Balestra et al., 2012), which would, however, also need a different experimental procedure. Additionally, neuropsychological measures are generally susceptible to changes in the strategic behavior, motivational drive, and more specifically, practice effects in executive function tests have been shown to vary between test subjects and depend on many factors (Calamia et al., 2012). Thus, given this limitation, it would be worthwhile for future research to combine computer-based approaches to study behavioral and longer-lasting effects with parallel recordings of objective measures such as CFFF and neuroimaging methods in extreme environments. This might be useful to capture a sophisticated view of human cognitive performance and brain cortical function beyond that what can be revealed by each method alone. Lastly, other executive functions that were not captured by our tests could be measured along other aspects such as the interactive effects of increased or decreased partial pressure of oxygen, nitrogen, and carbon dioxide (e.g., exercise) on executive functions (Freiberger et al., 2016; Brebeck et al., 2017) and the role of diving experience given our heterogeneous sample regarding diving experience. However, based on our experimental findings of selective executive function impairments by an approach that was the first within this field of research, more specific theory-driven hypothesis could be derived in future studies, e.g., to test specific other important functions that are associated with ACC activity such as emotional regulations (Bush et al., 2000), which are known to be influenced by nitrogen narcosis (Jurado and Rosselli, 2007; Löfdahl et al., 2013).

## Ethics Statement

This study was carried out in accordance with the recommendations of ‘Ethical Principles of Psychologists and Code of Conduct, APA, Deutsche Gesellschaft für Psychologie’ with written informed consent from all subjects. All subjects gave written informed consent in accordance with the Declaration of Helsinki. The protocol was approved by the ethics committee of the ‘Deutsche Gesellschaft für Psychologie’.

## Author Contributions

FS contributed to the design of the study, performed the data acquisition, analysis, interpreted the data and wrote the manuscript. MD substantially contributed to the design of the study, data analysis and interpretation. Moreover, MD critically revised the manuscript and approved the final versions and its content.

## Conflict of Interest Statement

The authors declare that the research was conducted in the absence of any commercial or financial relationships that could be construed as a potential conflict of interest.
